# Water table depth and plant species determine the direction and magnitude of methane fluxes in floodplain meadow soils

**DOI:** 10.1002/ece3.11147

**Published:** 2024-03-10

**Authors:** Mike Peacock, Clare Lawson, David Gowing, Vincent Gauci

**Affiliations:** ^1^ Department of Geography and Planning, School of Environmental Sciences University of Liverpool Liverpool UK; ^2^ Department of Aquatic Sciences and Assessment Swedish University of Agricultural Sciences Uppsala Sweden; ^3^ School of Environment, Earth and Ecosystem Sciences Open University Milton Keynes UK; ^4^ Birmingham Institute of Forest Research University of Birmingham Birmingham UK; ^5^ School of Geography Earth and Environmental Science University of Birmingham Birmingham UK

**Keywords:** aerenchyma, ecosystem, greenhouse gas, hydrology, meadow, methane, plants, vegetation, water table, wetland

## Abstract

Methane (CH_4_) is a powerful greenhouse gas with ongoing efforts aiming to quantify and map emissions from natural and managed ecosystems. Wetlands play a significant role in the global CH_4_ budget, but uncertainties in their total emissions remain large, due to a combined lack of CH_4_ data and fuzzy boundaries between mapped ecosystem categories. European floodplain meadows are anthropogenic ecosystems that originated due to traditional management for hay cropping. These ecosystems are seasonally inundated by river water, and straddle the boundary between grassland and wetland ecosystems; however, an understanding of their CH_4_ function is lacking. Here, we established a replicated outdoor floodplain‐meadow mesocosm experiment to test how water table depth (45, 30, 15 cm below the soil surface) and plant composition affect CH_4_ fluxes over an annual cycle. Water table was a major controller on CH_4_, with significantly higher fluxes (overall mean 9.3 mg m^−2^ d^−1^) from the high (15 cm) water table treatment. Fluxes from high water table mesocosms with bare soil were low (mean 0.4 mg m^−2^ d^−1^), demonstrating that vegetation drove high emissions. Larger emissions came from high water table mesocosms with aerenchymatous plant species (e.g. *Alopecurus pratensis*, mean 12.8 mg m^−2^ d^−1^), suggesting a role for plant‐mediated transport. However, at low (45 cm) water tables *A. pratensis* mesocosms were net CH_4_ sinks, suggesting that there is plasticity in CH_4_ exchange if aerenchyma are present. Plant cutting to simulate a hay harvest had no effect on CH_4_, further supporting a role for plant‐mediated transport. Upscaling our CH_4_ fluxes to a UK floodplain meadow using hydrological modelling showed that the meadow was a net CH_4_ source because oxic periods of uptake were outweighed by flooding‐induced anoxic emissions. Our results show that floodplain meadows can be either small sources or sinks of CH_4_ over an annual cycle. Their CH_4_ exchange appears to respond to soil temperature, moisture status and community composition, all of which are likely to be modified by climate change, leading to uncertainty around the future net contribution of floodplain meadows to the CH_4_ cycle.

## Graphical abstract

Using a combination of field measurements, mesocosm experiments, and hydrological modelling, we show that UK floodplain meadows are net emitters of the powerful greenhouse gas methane. These emissions are driven by high water tables and plant species with arenchymatous tissue.

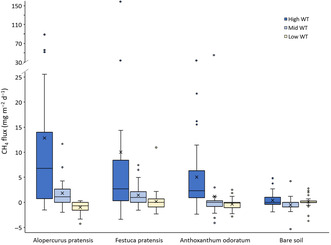

## 1 INTRODUCTION

Methane (CH_4_) is a powerful greenhouse gas and an important driver of climatic warming. Ecosystems are fundamental components of the global CH_4_ budget; oxic soils act as moderate sinks of 34 Tg year^−1^ (6% of all uptake), while wetlands are large sources, emitting 180 Tg year^−1^ (~30% of all emissions) (Saunois et al., 2020) (while simultaneously sequestering climate‐cooling volumes of carbon; Neubauer & Verhoeven, 2019). Wetland sources and soil sinks are both uncertain however, and more observations of CH_4_ fluxes *and* accompanying parameters are needed to constrain the global budget (Lan et al., 2021), which is a key step towards CH_4_ mitigation and achieving the goals of the Paris Agreement (Nisbet et al., 2020).

Some ecosystems (e.g. lakes, bogs) can be neatly placed into categories, and relatively accurate assumptions about their net CH_4_ emissions can be made due to a long history of research (e.g. see Ehhalt, 1974, and Harriss et al., 1985, for early studies of lakes and peatlands, respectively). Other ecosystems transgress boundaries, and floodplain meadows are one such example. Temperate floodplain meadows are classified at a European level as “moist or wet eutrophic and mesotrophic grassland” or “species‐rich lowland flood meadows” (respective EUNIS habitats E3.4 and E2.14; EEA, 2019). These semi‐natural ecosystems developed in northern Europe due to long‐term management for hay production for livestock (Rothero et al., 2020). Traditionally, vegetation was allowed to grow tall in spring and harvested for hay in summer to provide animal fodder. After the hay cut, livestock would be grazed, preventing re‐growth of taller species, and resulting in flower‐rich meadows (Rothero et al., 2016). The presence/absence and duration of flooding in these meadows now depends on human management and environmental conditions. As such, the soil water table will vary throughout the year, and floodplain meadows will uniquely fit into the contrasting categories of oxic soils or wetlands accordingly. These fluctuations in water table will have a direct effect on CH_4_ fluxes (Evans et al., 2021), with higher CH_4_ production/lower CH_4_ consumption under wet conditions (Segers, 1998). Additionally, water table will control plant species, survival and biomass (Gattringer et al., 2018) and this in turn will indirectly affect CH_4_ fluxes, either via plant‐mediated CH_4_ transport through aerenchymatous tissues or by the supply of labile methanogenic substrates to the soil (Whalen, 2005). Thus, the magnitude of CH_4_ fluxes and source/sink dynamics will be interactively driven by water table and plant species. This phenomenon has been well explored in other ecosystems, particularly peatlands (e.g. Bubier et al., 1995; Roulet et al., 1993; Shannon & White, 1994) and wetlands on mineral soils (e.g. Bartlett et al., 1989; Grünfeld & Brix, 1999; Tanner et al., 1997). However, there is a lack of CH_4_ data from floodplain meadows which are necessary both to reduce the uncertainty in regional and global budgets (Sun et al., 2013), and to allow a complete understanding of floodplain meadow ecosystem services to be achieved (see Lawson et al., 2018). This need is particularly relevant for the UK, where numerous floodplain meadow restoration projects have been ongoing since 2000 (Rothero et al., 2020). Here, we report on an investigation of CH_4_ fluxes from floodplain meadow soils. We first used a factorial mesocosm experiment to test whether the magnitude and direction of CH_4_ fluxes, measured over a full year, depends on depth to water table and vegetation composition. Second, we conducted snapshot CH_4_ flux measurements in a UK floodplain meadow during the growing season to compare with our mesocosm fluxes. Finally, we upscaled our fluxes using long‐term water table modelling from a floodplain meadow to evaluate how annual emissions and source/sink behaviour varied with hydrology.

## 2 METHODS

### 2.1 Mesocosm experimental design

**FIGURE 1:**
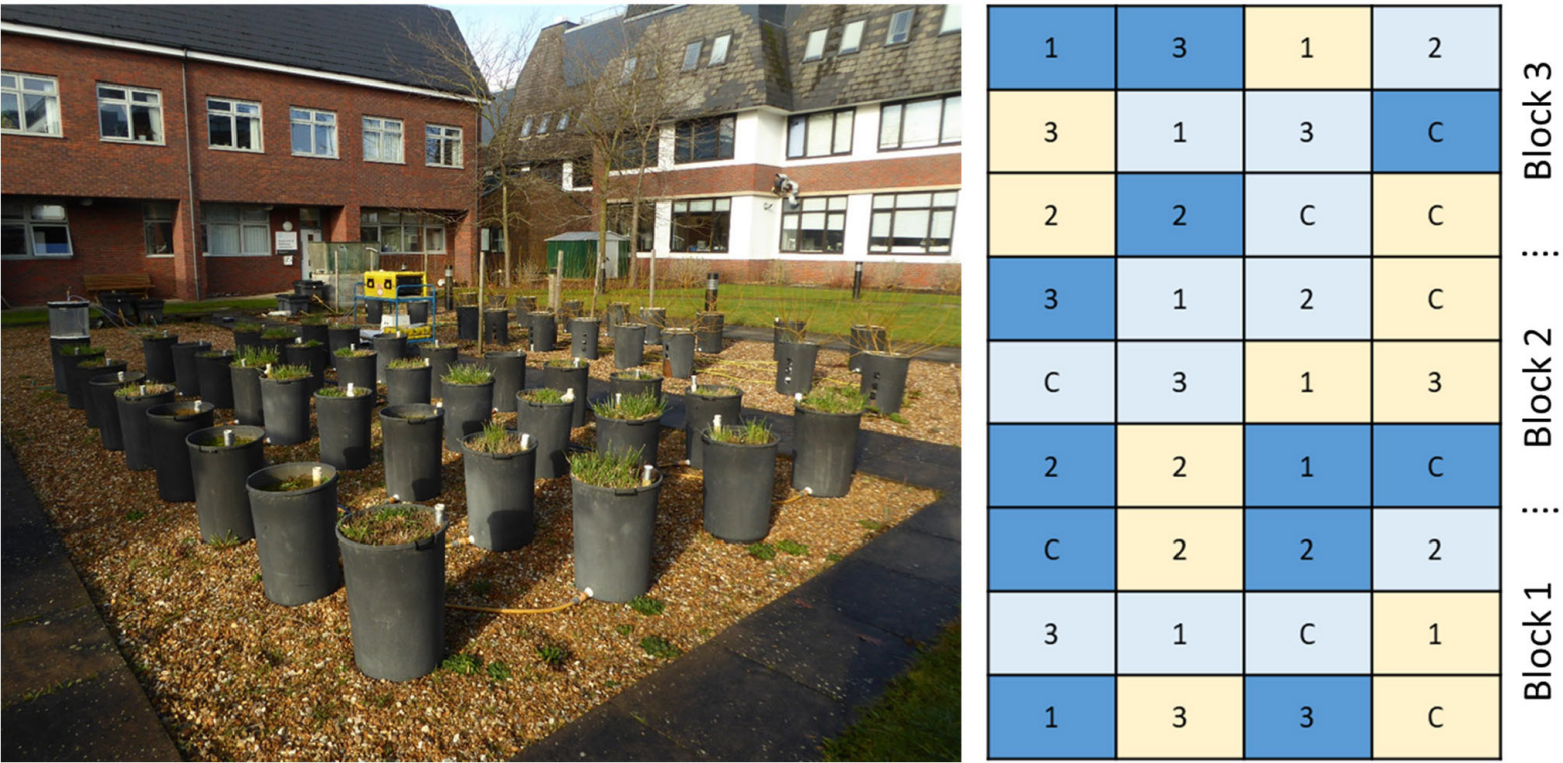
Left panel: the 36 mesocosms during February 2017. In the centre is the yellow Los Gatos Fast Methane Analyser, connected to a static chamber on the top left mesocosm. Behind the analyser is the grey central reservoir tank, and left of this are several black boxes, which are control float chambers to regulate water tables. The mesocosms also contain dipwells, which were used to monitor the water table height in this experiment. Note that the mesocosms in the background containing willow trees belong to a different experiment. Right panel: schematic of the mesocosm array (36 mesocosms) showing the three blocks, with randomised treatments within each block. Numbers 1, 2, 3 refer to vegetation assemblages: 1. *F. pratensis*, *L. pratensis*, *F. ulmaria*, *P. lanceolata*, 2. *A. odoratum*, *L. corniculatus*, *P. vulgaris*, *L. autumnalis*, 3. *A. pratensis*, *T. pratense*, *S. officinalis*, *C. nigra*. C represents the bare soil control mesocosms. Colors represent water table treatments below soil surface: 15 cm (dark blue), 30 cm (pale blue), and 45 cm (pale yellow).

We established a mesocosm experiment at the Open University, UK (52.02567, −0.70819), in March 2015. The climate is temperate with mean annual temperature of 10.3°C and mean total annual precipitation of 652 mm (1991–2020 data from the UK Met Office Woburn station, 7.5 km away). We used the same mesocosm array developed by Araya et al. (2010), which consisted of 36 opaque, cylindrical polyvinyl chloride mesocosms, arranged in a 4 × 9 grid (Figure 1). Each mesocosm had a diameter of 36 cm and a height of 55 cm. The mesocosms were filled with layers of gravel, sand, and sandy loam soil in March 2015 and aimed to mimic the well‐structured soils of floodplain meadows. Gravel filled the bottom 5 cm of each mesocosm to allow the incoming water from the control chamber to disperse evenly. On top of the gravel, 3 cm of sand (with a uniform particle size of 225 μm, WBB Minerals® RH65) was placed followed by the sandy loam soil which came from Rothamsted Research's experimental farm at Woburn, Bedfordshire (and we assume this transferred the existing microbial population into the mesocosms). Each layer of soil was separated using a porous membrane to allow incoming water to disperse evenly across the mesocosm and to facilitate precise regulation of water table. Soil samples from all mesocosms to a depth of 20 cm were taken in May 2015 before planting and experimental work began: mean soil pH was 6.6, Olsen extractable soil phosphorus was 74.5 mg/kg, and soil organic carbon content was 1.3%. Drainable porosity was 15% and was calculated from soil moisture release curves as the volume of water lost when a sample is taken from saturation down to field capacity (a tension equivalent to a water table depth of 50 cm; and note that throughout we use positive values of water table depth to refer to a water table below the soil surface). 10% air‐filled porosity (AFP) is the threshold for oxygen to diffuse effectively within the soil and soils with <10% AFP are vulnerable to anoxia. We calculated a threshold water table depth of 39.2 cm for anoxia, equating to 10% AFP in the root zone (top 20 cm of the soil profile) (Taylor, 1950).

After establishment, there was a ~15 month settling period, during which time we assume some additional microbial colonisation of the mesocosms occurred (e.g. via faunal vectors, atmospheric deposition). Mesocosms were then planted with different vegetation species in June 2015 to give three vegetation assemblages (nine mesocosms per vegetation assemblage), chosen to be representative of UK floodplain meadow plant communities (i.e. MG4 *Alopecurus pratensis* – *Sanguisorba officinalis* mesotrophic grassland in the UK National Vegetation Classification; see Table S1 and Prosser et al., 2023). A fourth control vegetation treatment was established by leaving nine mesocosms unplanted (i.e. bare soil). Each assemblage contained one grass, one legume and two forbs. Assemblages were selected based on functional traits; specific leaf area (SLA) and plant height, data on which was extracted from the TRY database (Kattge et al., 2020). The three assemblages were as follows:

1. *Festuca pratensis*, *Lathyrus pratensis*, *Filipendula ulmaria*, *Plantago lanceolata* (SLA < 25 mm^2^ mg^−1^, height 20–80 cm).
2. *Anthoxanthum odoratum*, *Lotus corniculatus*, *Prunella vulgaris*, *Leontodon autumnalis* (SLA > 25 mm^2^ mg^−1^, height < 25 cm).
3. *Alopecurus pratensis*, *Trifolium pratense*, *Sanguisorba officinalis*, *Centaurea nigra (*SLA > 25 mm^2^ mg^−1^, height > 40 cm).

Seeds of these species were sown on seed trays in January 2015 and chilled for a period of 8 weeks before being moved into a growth room (20°C day, 10°C night, humidity 65%, 13 h daylight). Seedlings were transferred to larger pots and transferred outside in May 2015 to acclimatise. Three plants of the four species in each assemblage were planted in June 2015. The plants were allowed to become established in the pots under free‐draining conditions before the three water table treatments were imposed in October 2015.

All mesocosms were connected by hoses to one of three control float chambers, which were connected to a central reservoir tank (volume 1200 L) filled with tap water. The three 18 L control float chambers were elevated at different heights and used to maintain three different water tables (12 mesocosms per water table) which were 45, 30, and 15 cm below the soil surface, and which we refer to respectively as low, mid, and high water tables. Note that water table within each mesocosm could be manipulated independent of its neighbours (refer to Araya et al., 2010 for further details of the mesocosm array, including photos and diagrams). Nine mesocosms of each water table treatment were planted, while three remaining mesocosms per water table treatment served as bare soil controls and contained no plants. This factorial approach (3× water tables, 4× vegetation assemblages) resulted in a total of three mesocosms per each individual treatment. The mesocosm array was split into three blocks, and the three replicates of each individual treatment were placed one per block, with locations within blocks randomised (Figure 1).

Floodplain meadows are often intensively managed by cutting/mowing, traditionally to provide a midsummer hay harvest but also to promote species richness (Gerard et al., 2008; Rothero et al., 2020). A second cut during autumn is sometimes done, which has the additional benefit of reducing flood‐derived nutrient loads (Bowskill et al., 2023). We simulated these management interventions by cutting all vegetation down to 4 cm on two occasions; first at the end of June 2016, and again in mid‐November 2016.

### 2.2 Mesocosm CH_4_ flux measurements

CH_4_ fluxes were measured during daylight hours on 10 occasions between 2 May 2016 and 11 April 2017, on an approximate monthly basis, with the exception of December and January when no measurements were made. Fluxes were measured using a cylindrical static chamber of 36 cm diameter (the same diameter as the mesocosms) and 42 cm height, constructed from reinforced transparent sheets of fluorinated ethylene propylene film attached to a cylinder of wire mesh, and with a polymethylmethacrylate top fitted with two gas sampling ports. During flux measurements, gas impermeable tubing was attached to the ports and used to circulate air between the chamber and a CH_4_ analyser measuring real‐time concentrations. Two different Los Gatos Research analysers were used during the course of the study: an RMA200 Fast Methane Analyser and an Ultraportable Greenhouse Gas Analyser. Both analysers use the same technology (cavity‐ring down laser spectroscopy) and have precision <1 ppb CH_4_. Flux measurements were made until a linear change in CH_4_ concentration within the chamber was observed, which was typically <5 min, although on some occasions no linear change was apparent. Fluxes were calculated using a linear regression between chamber closure time and CH_4_ mass, and CH_4_ was adjusted for air temperature and pressure, which were measured with a Commeter C4141 probe. Fluxes were accepted if the linear regression was significant (*p* < .05) regardless of the *R* ^2^ value (as in Peacock et al., 2017). Non‐significant fluxes were categorised as zero fluxes (*n* = 101) and were retained in the dataset for analysis.

### 2.3 Field design

We conducted snapshot CH_4_ flux measurements on 7 June 2016 at Cricklade North Meadow National Nature Reserve (51.649, −1.864), a temperate UK floodplain meadow (see site photo, Figure S1). Mean annual temperature is 10.2°C, and mean total annual precipitation is 823 mm (1991–2020 data from the UK Met Office Cirencester station, 10 km away). The hydrological properties of the field soil are similar to that of the mesocosm soil; the drainable porosity is 12% and the anoxia threshold water table depth is 34.1 cm. Three locations across the meadow were selected for CH_4_ measurements. Within each location, the vegetation was sampled in five 1 m × 1 m quadrats and the presence of all vascular plants and bryophytes were recorded. The field locations included the same species as used in the mesocosm plantings, and all locations were classed as MG4 (*Alopecurus pratensis*–*Sanguisorba officinalis* mesotrophic grassland) (see Table S1 for the presence/absence data of species). However, the three locations split along a hydrological gradient into different subcommunities: MG4a *Dactylis glomerata*, MG4b Typical and MG4d *Agrostis stolonifera*. These three subcommunities typically follow a hydrological gradient where MG4a is frequently dry and rarely inundated with floodwater, MG4b is intermediate, and MG4d is often waterlogged even during the growing season (Prosser et al., 2023). Water tables were modelled for each individual quadrat using an analytical solution to soil‐drainage equations, and seven dipwells across site were used to validate the spatial hydrological model (the model and its validation are described in Gowing et al., 1998), and the results supported the hydrological separation of subcommunities, with respective mean annual water tables (Jan 2010 to Dec 2015, see Figure S2) of 0.41, 0.37 and 0.25 m for the three locations which we hereafter refer to as “deep” (MG4a), “mid” (MG4b) and “shallow” (MG4d). Long‐term water table behaviour was complex, however, and at times the water table at the mid location is deeper in the soil than the deep location (Figure S2). This complexity is reflected in the fact that on the day that CH_4_ flux measurements took place, mean water tables were, respectively, −0.58, −0.60 and −0.25 m for the deep, mid and shallow locations.

### 2.4 Field CH_4_ flux measurements

Field CH_4_ measurements took place between 10:00 and 14:00. Mean air temperature during sampling was 27°C. Fluxes were measured with a Los Gatos Research Ultraportable Greenhouse Gas Analyser connected to an acrylic static chamber, with dimensions 0.6 m × 0.6 m × 0.8 m (length × width × height). The chamber was covered in reflective silver foil to minimise heating. Because of the snapshot nature of the sampling, no collars were installed into the soil. Instead, the chamber was gently pushed into the soil. At each of the three locations (deep, mid and shallow), five chamber measurements were made. Flux measurements and data processing were the same as described in Section 2.2. Note that field measurements used a reflective/dark chamber, while mesocosm measurements used a clear/light chamber, and thus the results from the two may not be truly comparable because dark chambers can cause changes to internal gas transport (and therefore CH_4_ flux) in some plant species (Günther et al., 2014).

### 2.5 Upscaling mesocosm CH_4_ fluxes with field water tables

We used the long‐term modelled water tables (Figure S2) from Cricklade North Meadow to upscale our mesocosm CH_4_ fluxes in order to determine how hydrology might affect the annual budgets and source/sink behaviour of floodplain meadows. To do this, for each quadrat (*n* = 15) with modelled water table depths, we first calculated the mean number of weeks where anoxia theoretically developed (see Sections 2.1 and 2.4). Second, we divided the year into two periods using a threshold soil temperature of 5°C for grass growth (Hopkins, 2000). For Cricklade North Meadow, these periods broadly equate to Nov to March being <5°C (“cold”) and Apr to Oct being >5°C (“warm”). This approach thus gave four combinations of soil conditions: cold anoxic, cold oxic, warm anoxic, warm oxic. For each condition, we allocated a CH_4_ emission factor (EF) based on the mesocosm results and multiplied these by the respective periods of time that each quadrat experienced each condition (see Section 3.3) to calculate annual budgets.

### 2.6 Statistical analysis

Statistical analyses were done using IBM SPSS Statistics 29. The field CH_4_ flux data were normally distributed but for the mesocosm experiment, CH_4_ flux data were not normally distributed, and data transformations failed to achieve normality. However, F tests are relatively robust to non‐normal data (Blanca Mena et al., 2017) and therefore we used an ANOVA to test for treatment effects on mesocosm CH_4_ flux, using water table, vegetation assemblages and sampling date as fixed factors (although note that data here are temporally pseudoreplicated). For the field CH_4_ flux data, we used an ANOVA to test for a significant effect of location (deep vs. mid vs. shallow). Tukey HSD tests were used for all *post‐hoc* comparisons. We used Spearman correlation to test for a relationship between mesocosm CH_4_ flux and air temperature. All differences were considered significant when *p* < .05. Errors are given as standard errors of the mean (SEM). To calculate annual fluxes, we linearly interpolated between sampling dates.

## 3 RESULTS

### 3.1 Mesocosm CH_4_ flux measurements

**FIGURE 2:**
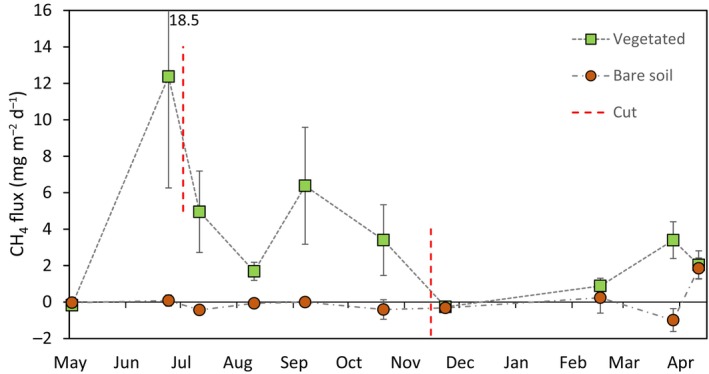
Mean CH_4_ fluxes ± SEMs for all vegetated mesocosms (*n* = 27) and bare soil mesocosms (*n* = 9) for all sampling dates. Dashed red lines indicate when vegetation was cut to simulate mowing. ANOVA shows a significant effect of sampling date on CH_4_ flux (*F* = 2.93, *p* = .003).

**FIGURE 3:**
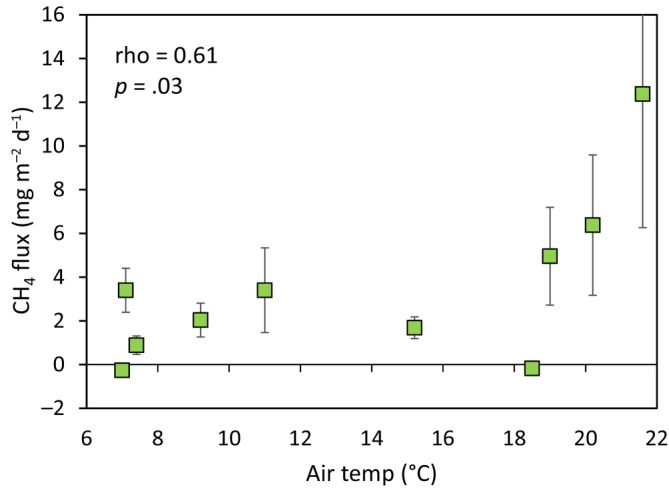
Scatter plot showing the significant correlation between mean air temperature and mean CH_4_ fluxes ± SEMs from vegetated mesocosms (*n* = 27) for all 10 sampling dates.

Overall mean CH_4_ flux for the entire study period was 2.6 ± 0.6 mg CH_4_ m^−2^ d^−1^ (*n* = 360). Fluxes from the bare soil control mesocosms were mostly zero or slightly negative, with an overall study mean of zero, in contrast to 3.47 ± 0.8 mg CH_4_ m^−2^ d^−1^ from vegetated mesocosms (Figure 2). The highest individual flux was 160 mg CH_4_ m^−2^ d^−1^ from a *F. pratensis* mesocosm during June. There was significant variation in CH_4_ flux between sampling dates (*F* = 2.93, *p* = .003, Figure 2) with higher fluxes during the summer months, and there was a significant correlation between air temperature and flux (Figure 3).

**FIGURE 4:**
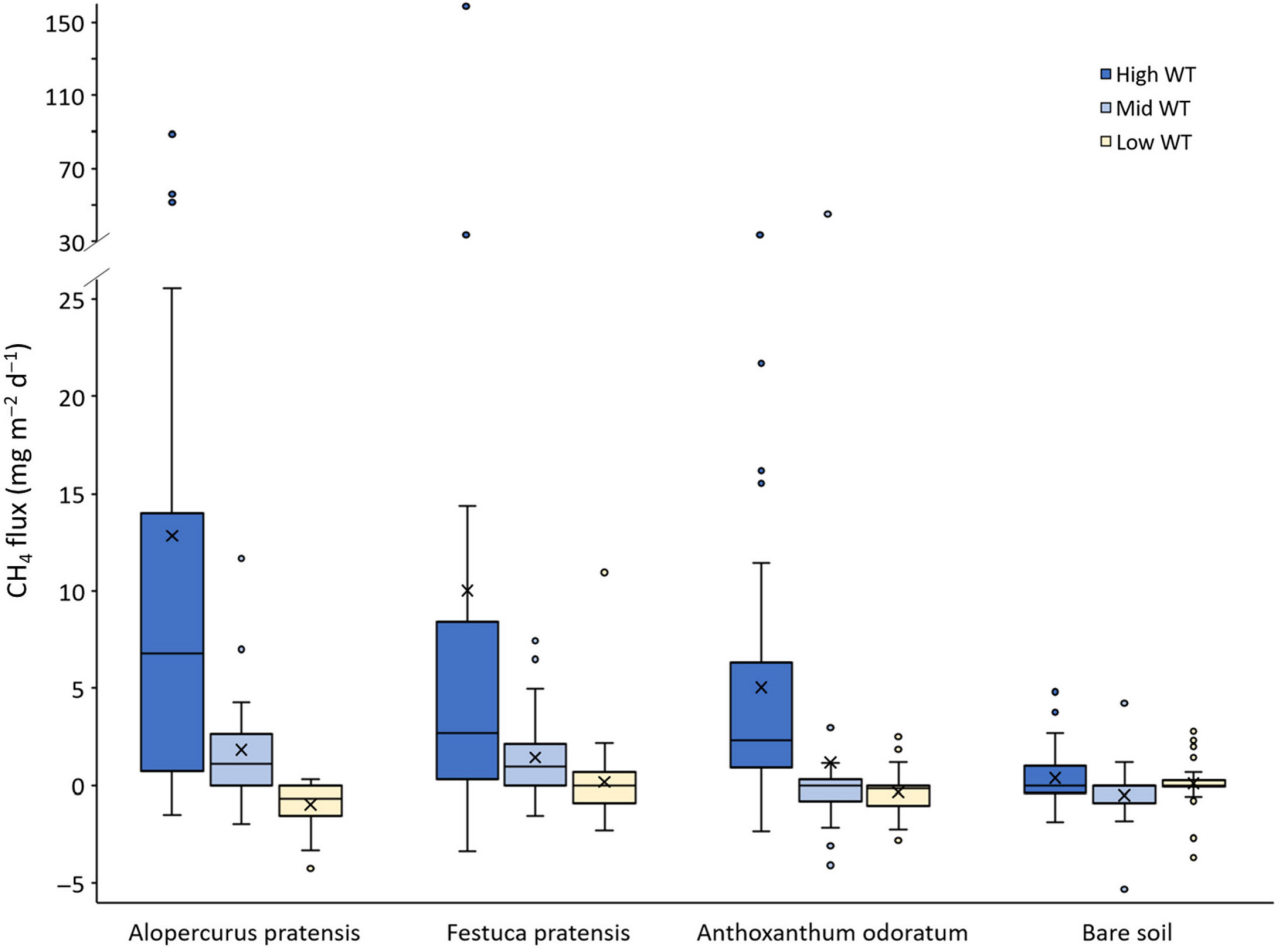
Box plot of mesocosm CH_4_ fluxes for the three vegetation assemblages, plus bare soil controls, grouped by water table (each bar represents three mesocosms, measured on 10 occasions). Water tables are 15 (high), 30 (mid) and 45 cm (low) below the soil surface. Boxes represent medians and interquartile range (IQR), whiskers mark minimum and maximum values, excluding outliers (calculated as box limits ±1.5 × IQR). Also shown are mean fluxes (x) and outliers (o). ANOVAs show significant effects for water table (*F* = 18.7, *p* = .001), vegetation (*F* = 3.86, *p* = .01) and the interaction between water table and vegetation (*F* = 2.87, *p* = .01). For water table, Tukey HSD tests are significant for high versus low and high versus mid (*p* < .001). For vegetation, Tukey HSD tests are significant for bare soil versus *Alopercurus pratensis* (*p* = .012) and bare soil versus *Festuca pratensis* (*p* = .046). Figure S3 shows the same data grouped by individual mesocosm.

There were large differences in CH_4_ flux between treatments and these were significant for water table (*F* = 18.7, *p* < .001), vegetation (*F* = 3.86, *p* = .01) and the interaction between water table and vegetation (*F* = 2.87, *p* = .01) (Figure 4, Figure S3). Fluxes were greatest under high (15 cm) water tables, and from high water table mesocosms with the *A. pratensis* and *F. pratensis* plant assemblages. In general, smallest fluxes were from bare soil mesocosms and low (45 cm) water tables. However, the greatest mean CH_4_ uptake (−0.98 mg CH_4_ m^−2^ d^−1^) was for the *A. pratensis* assemblage under low water table (Figure 4), and this was significantly different (*F* = 3.32, *p* = .022) than low‐water table fluxes from bare soil (0.10 mg CH_4_ m^−2^ d^−1^, *p* = .048) and *F. pratensis* mesocosms (0.18 mg CH_4_ m^−2^ d^−1^, *p* = .03).

Overall mean annual flux was 0.85 g CH_4_ m^−2^ year^−1^, or 1.14 g CH_4_ m^−2^ year^−1^ when only vegetated mesocosms were considered. The greatest and smallest annual fluxes from any treatment were both found for *A. pratensis* assemblages: 4.23 and −0.35 g CH_4_ m^−2^ year^−1^ for the high (15 cm) and low (45 cm) water table treatments respectively (Table 1).

**TABLE 1:** Mean annual mesocosm CH_4_ fluxes for all treatments, grouped by vegetation assemblage and water table.

	CH_4_ flux (g m^2^ year^−1^)		
	High	Mid	Low
*Alopercurus pratensis*	4.23	0.54	−0.35
*Festuca pratensis*	3.37	0.47	0.04
*Anthoxanthum odoratum*	1.59	0.48	−0.11
Bare soil	0.16	−0.21	0.03

*Note*: Water tables are 15 (high), 30 (mid) and 45 cm (low) below the soil surface.

### 3.2 Field measurements

**FIGURE 5:**
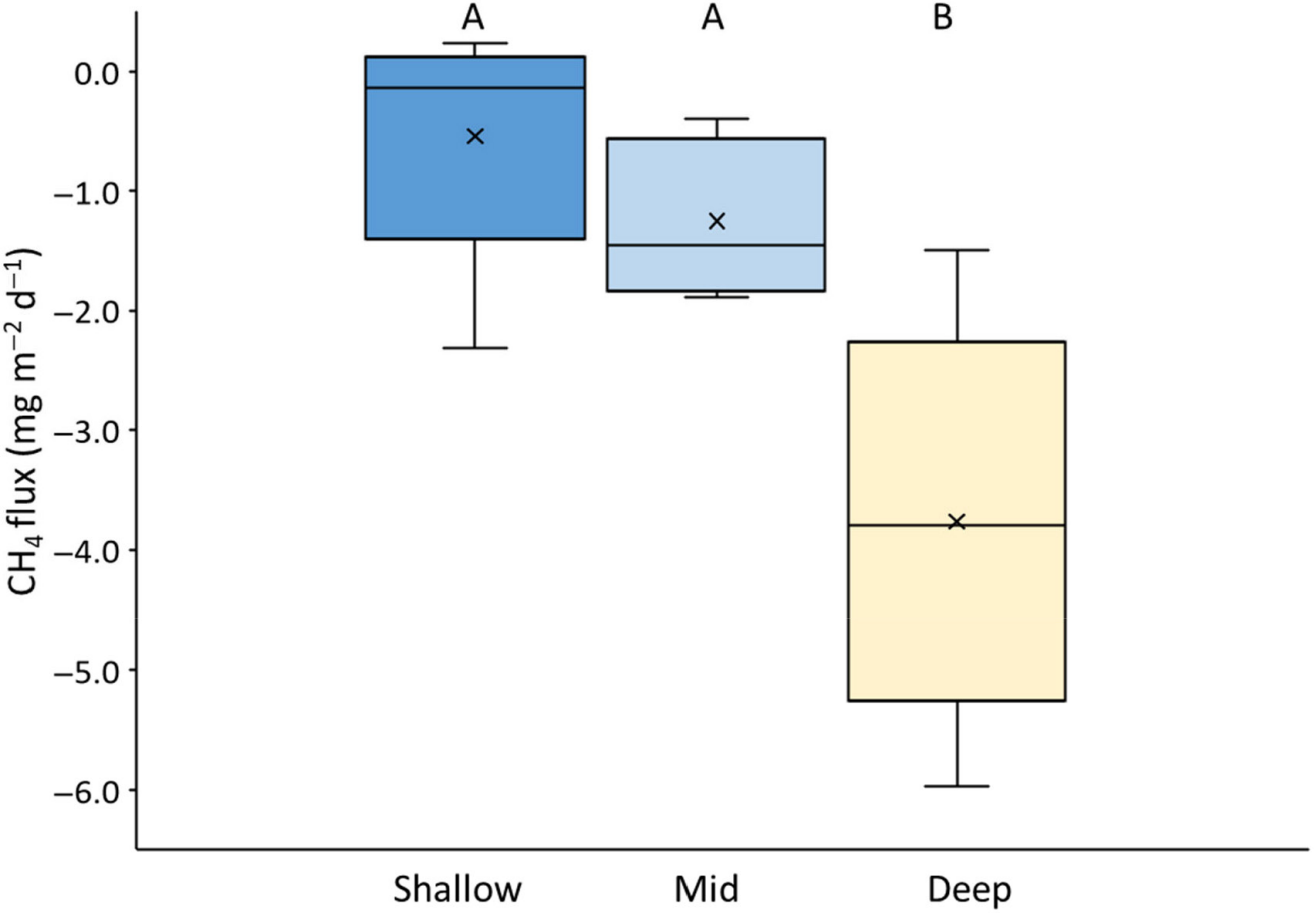
Box plot of field CH_4_ fluxes for the three locations with different water tables (for each bar, *n* = 5). Boxes represent medians and interquartile range (IQR), whiskers mark minimum and maximum values, excluding outliers (calculated as box limits ±1.5 × IQR). Also shown are mean fluxes (x). ANOVA shows significant effects for water table (*F* = 10.1, *p* = .003). Tukey HSD tests are significant for shallow versus deep (*p* = .003) and mid versus deep (*p* = .015), and significant differences are marked by letters on the panel. Mean annual water tables are 25, 37 and 41 cm for the shallow, mid and deep locations.

Fluxes measured during early June 2016 at Cricklade North Meadow were the same magnitude as the mesocosm fluxes, but were mostly negative due to the water tables being deeper in the field than in the mesocosms (Figure 5). In line with the mesocosm results (Figure 4), there was a significant effect of water‐table depth on CH_4_ flux, with CH_4_ uptake being greatest in the driest part of the floodplain meadow. Air temperature during field sampling (27°C) was higher than air temperature during any of the mesocosm flux measurements (maximum = 22°C in late June).

### 3.3 Upscaling mesocosm CH_4_ fluxes with field water tables

We used the results from Section 3.1 to set the EFs for the four soil conditions: cold anoxic, cold oxic, warm anoxic, warm oxic. Results from the mesocosms showed higher CH_4_ emissions under warmer temperatures, with fluxes being notably higher at temperatures ≥19°C (Figures 2, 3). We therefore calculated the warm anoxic EF as the mean CH_4_ flux from all high water table (15 cm) vegetated mesocosms during the June to Oct period (15.2 ± 4.1 mg CH_4_ m^−2^ d^−1^), and the cold anoxic EF as the mean flux from the same mesocosms for Nov–May (3.43 ± 0.7 mg CH_4_ m^−2^ d^−1^) (Figure 2). Using the high water table (15 cm) flux, rather than the mid water table (30 cm, which is also above the mesocosm anoxic depth of 39 cm) may be thought to produce an overly generous EF. However, we assume this is not the case, because water tables in the field frequently rise higher than 15 cm, and surface flooding is common during winter (Figure S2). These flood events will likely lead to large spikes in CH_4_ emission, significantly larger than anything measured in the mesocosms; thus, we assume that an EF based on the 15 cm water table treatment is appropriate.

There was no significant relationship (rho = 0.43, *p* = .21) between temperature and CH_4_ flux when only the low water table (45 cm) mesocosms were considered, suggesting that CH_4_ uptake was not controlled by temperature (Figure S4). We therefore used the same EF for cold oxic and warm oxic conditions, which we calculated as the mean CH_4_ flux for all vegetated, low water table (45 cm) mesocosms (−0.38 ± 0.2 mg CH_4_ m^−2^ d^−1^). 45 cm is below the anoxic depth for the mesocosms, and thus we assume this EF is appropriate for modelling oxic emissions in the field. Soils experienced anoxia for 39%–56% of the year, and the CH_4_ emissions during these periods were greater than the oxic‐period CH_4_ uptake, resulting in all three locations across the floodplain meadow acting as annual net sources of CH_4_, with respective emissions of 0.8, 1.1 and 1.5 g CH_4_ m^−2^ year^−1^ (Table 2), giving a mean of 1.14 g CH_4_ m^−2^ year^−1^ (note that overall mean from the vegetated mesocosms was also 1.14 g CH_4_ m^−2^ year^−1^).

**TABLE 2:** Total number of days in a year that each of the three locations (deep, mid and shallow water tables) at Cricklade North Meadow experienced different soil conditions (anoxic cold, anoxic warm, and oxic), along with emission factors for each condition, and total CH_4_ emissions from each location under each condition.

Number of days	Anoxic cold	Anoxic warm	Oxic cold + warm	Total
Deep WT	106	36	223	365
Mid WT	132	49	184	365
Shallow WT	132	71	162	365
EF (mg CH_4_ m^−2^ d^−1^)	3.43	15.2	−0.38	—
Flux for period (mg CH_4_ m^−2^)				
Deep WT	363	546	−84	825
Mid WT	452	746	−70	1128
Shallow WT	451	1075	−61	1465

*Note*: Mean annual water tables are 25, 37 and 41 cm for the shallow, mid and deep locations.

## 4 DISCUSSION

In our controlled, replicated mesocosm experiment, we found methane fluxes varied throughout the year, and were greatest in summer when air temperatures were higher, likely due to higher rates of methanogenesis within the soils (Segers, 1998). We found significant effects of both water table and vegetation composition on CH_4_ fluxes. Our field measurements supported a primary role of water‐table depth on controlling emissions, and hydrological modelling and upscaling showed that high CH_4_ emissions during periods of anoxia drive the annual CH_4_ balance of floodplain meadows. Below, we discuss our findings in more detail.

### 4.1 Effect of water table on mesocosm fluxes

Emissions of CH_4_ were greatest from mesocosms with high water tables (15 cm below the soil surface), than those with mid (30 cm) or low (45 cm) water tables. The relationship between water table and CH_4_ is entirely as expected, because wetter, anoxic conditions favour CH_4_ production and hinder CH_4_ oxidation (Segers, 1998). Other studies have shown a switch from zero/negative CH_4_ fluxes to positive emissions at 25–30 cm water table depth in riparian wetlands, forested wetlands, and managed peatlands (Audet, Johansen, et al., 2013; Evans et al., 2021; Hondula et al., 2021), although the exact threshold will depend on soil structure (Askaer et al., 2010). These fluxes then continue to increase as water tables rise towards the soil surface. Fluxes were frequently negative or around zero in our low water table (45 cm) treatment, while at mid water tables (30 cm) fluxes were generally positive, but CH_4_ uptake did occasionally occur (this is particularly evident in the negative error bar crossing zero for *A. odoratum* in Figure 4). Thus, our data support the idea for a threshold water table ~15 to 20 cm in well‐structured soils. Our measured mesocosm fluxes were in the same range as other studies from temperate riparian wetlands and grasslands (Ambus & Christensen, 1995; Audet, Johansen, et al., 2013; Itoh et al., 2007; Sha et al., 2011; Sun et al., 2013) although our highest fluxes (max = 160 mg CH_4_ m^−2^ d^−1^) were modest when compared to field‐measured values that sometimes reach as high as from 1000 to 3000 mg CH_4_ m^−2^ d^−1^ (Audet, Johansen, et al., 2013; Sha et al., 2011). However, we note that our high water table depth of 15 cm is still relatively deep when compared to some studies, and to our field site where prolonged inundation sometimes occurs (Figure S2), which may explain this difference. Finally, the category of “riparian wetland” will include a diverse array of soil types, nutrient statuses, and plant species compositions, all of which will make direct comparisons of CH_4_ flux between studies somewhat problematic. To aid comparisons future studies should measure the drainable porosity of their soil, which will likely be a dominant variable determining CH_4_ flux for a given water‐table depth.

### 4.2 Effect of vegetation on mesocosm fluxes

Fluxes of CH_4_ from our bare soil control mesocosms were low, even under high water tables, where the mean was just 0.4 mg CH_4_ m^−2^ d^−1^ (demonstrating that methanogens were present). In contrast to this, fluxes from high water table (15 cm) vegetated mesocosms were considerably greater (9.3 mg CH_4_ m^−2^ d^−1^). Our experimental design thus suggests that high CH_4_ emissions from our floodplain meadow soils are driven by the interaction between water table and plant species composition (and, presumably, their associated microbial communities). Significantly higher fluxes were found for two of the vegetation assemblages: the *A. pratensis* group (also including *Trifolium pratense*, *Sanguisorba officinalis* and *Centaurea nigra*) and *F. pratensis* group (also including *Lathyrus pratensis*, *Filipendula ulmaria* and *Plantago lanceolata*). There are two primary routes by which plants may influence CH_4_ emissions: (1) by providing labile substrates for CH_4_ production (Ström et al., 2012) and, (2) plants with aerenchymatous tissue can act as chimneys, enhancing emissions by transporting CH_4_ directly from the anoxic zone to the atmosphere (Greenup et al., 2000), or lowering emissions by transporting oxygen from the atmosphere to the rhizosphere, thereby suppressing CH_4_ production/increasing CH_4_ oxidation (Roura‐Carol & Freeman, 1999). There is some evidence to support a role of plant‐mediated emissions (Table 3). *A. pratensis* and *F. pratensis*, the grass species in the two high‐emitting vegetation assemblages, both have entire layers of aerenchymatous tissue (Wright et al., 2017), are deep‐rooted (Bowskill & Tatarenko, 2021), and have previously been shown to enhance CH_4_ emissions due to plant‐mediated transport (Przywara & Stêpniewska, 2002). In contrast the grass species (*Anthoxanthum odoratum*) in the vegetation assemblage with lower CH_4_ emissions does not form aerenchymatous tissue and is shallow‐rooted. Two other plant species in the low‐emitting vegetation assemblage, *Lotus corniculatus* and *Prunella vulgaris*, can form some aerenchymatous tissue, but not to the same extent as *A. pratensis* and *F. pratensis* (Wright et al., 2017) (Table 3). A further hint to the role of aerenchymatous tissue in controlling fluxes is that the *A. pratensis* mesocosms had the greatest CH_4_ uptake at low water tables (45 cm), with an annual flux of −0.35 g CH_4_ m^−2^ year^−1^, compared to −0.11 g CH_4_ m^−2^ year^−1^ for *A. odoratum*, and approximately zero for *F. pratensis* and bare soil. This suggests that there is plasticity in CH_4_ exchange if aerenchyma are present, and similar findings have been shown for tree emissions on tropical floodplains (Gauci et al., 2022). In floodplain meadows, low water tables are the natural state, with high water tables periodically interrupting this. Therefore, for the majority of the time in these ecosystems, deep‐rooted, aerenchymatous plants serve to enhance atmospheric methane removal.

**TABLE 3:** Table showing aerenchymatous tissue properties of the plant species in each vegetation assemblage and maximum rooting depth of species.

Assemblage	Species	Degree of aerenchymatous tissue	Rooting depth (cm)
Group 1	*Festuca pratensis*	High	160
	*Lathyrus pratensis*	None	135
	*Filipendula ulmaria*	Medium	40
	*Plantago lanceolata*	None	20
Group 2	*Anthoxanthum odoratum*	None	20
	*Lotus corniculatus*	Medium	170
	*Prunella vulgaris*	Medium	25
	*Leontodon autumnalis*	None	35
Group 3	*Alopecurus pratensis*	High	100
	*Trifolium pratense*	None	130
	*Sanguisorba officinalis*	None	190
	*Centaurea nigra*	None	240

*Note*: High = entire layer of aerenchymatous tissue, some = limited extent of aerenchymatous tissue/have the potential to develop some under certain conditions, none = no aerenchymatous tissue. References: Wright et al. (2017), Smirnoff and Crawford (1983), Poschlod et al. (2003), Kattge et al. (2020), Bowskill and Tatarenko (2021).

### 4.3 Vegetation cutting and mesocosm fluxes

European floodplain meadows are typically highly managed ecosystems, with mowing taking place during summer, and sometimes again during autumn (Bowskill et al., 2023). In our experiment, we simulated this management by cutting back vegetation once during summer, and again during autumn. Our experimental design (specifically, a lack of uncut controls) precludes us from performing statistical tests on the effects these cuts may have on CH_4_ flux, and post‐cut fluxes remained within the range of annual variation. Fluxes did decline to approximately zero following the November cut although any difference is equally likely to be driven by lower post‐cut temperatures reducing methanogenesis; mean air temperatures during the pre‐ and post‐cut sampling were 11°C and 7°C respectively. Lack of robust evidence for an effect of cutting provides support for plant‐mediated transport of CH_4_. In an experiment with *Eriophorum Vaginatum*, Greenup et al. (2000) found that cutting only decreased CH_4_ emissions when stems were cut below the water table surface, not above as was done in our study. Thus, our cutting left some standing vegetation as a pathway for CH_4_ diffusion from soil to atmosphere (Kelker & Chanton, 1997).

### 4.4 Field measurements and upscaling

Snapshot flux measurements during the growing season at Cricklade North Meadow were in agreement with the mesocosm data (Section 4.1) by also demonstrating a role of water‐table depth on CH_4_ fluxes. The greatest CH_4_ uptake was observed at the locations within the floodplain meadow where the mean annual water table was deepest (41 cm) and where oxic conditions prevailed for longest (223 days per year, compared to 184 days for the mid water table location). However, on the day of sampling the water table was essentially identical at the deep (58 cm) and mid (60 cm) locations. This difference in CH_4_ flux, despite no difference in instantaneous water table, suggests either a lag effect of water table on CH_4_ (e.g. Tangen & Bansal, 2019), or a role of plant species in modulated flux; note that plant communities did differ (Table S1) with the deep and mid communities being identified as MG4a and MG4b, respectively.

Modelling of hydrology suggested that the soil was likely to be anoxic for approximately half of the year. Upscaling mesocosm fluxes to the meadow showed that the relatively high CH_4_ emissions during these anoxic periods outweighed the oxic‐period CH_4_ uptake, and that all three locations in the floodplain meadow (which had mean annual water tables of 25, 37 and 41 cm) were net sources of CH_4_ on an annual basis. Other grassland and meadow studies have also found that seasonal flooding can dominate the annual CH_4_ budget (Antonijević et al., 2023; Chamberlain et al., 2015). Although acting as a net source, the magnitude of modelled emissions was modest; 0.8, 1.1 and 1.5 g CH_4_ m^−2^ year^−1^ at the deep (41 cm), mid (37 cm) and shallow (25 cm) water table sites, giving a mean of 1.14 g CH_4_ m^−2^ year^−1^. However, these upscalings are only approximate and could be refined in future by taking into account that plant communities also change across the floodplain meadow, and that these will affect the strength of CH_4_ sources and sinks, particularly where aerenchymatous species are abundant (see Section 4.2). However, mean annual modelled flux at Cricklade North Meadow was identical to those measured from the mesocosms (=1.14 g CH_4_ m^−2^ year^−1^) suggesting our modelling is robust.

### 4.5 Implications

The potential importance of carbon sequestration by floodplain meadows is widely recognised (Lawson et al., 2018). However, here we have shown that, depending on plant species and water table, these ecosystems can emit non‐trivial volumes of CH_4_, or serve to remove it from the atmosphere. Furthermore, our modelling suggests that, under current hydrological conditions, inundated periods lead to high emissions which overrides the non‐flood period CH_4_ sink, leading to net source behaviour. It is necessary to highlight that wetland CH_4_ emissions are a natural component of the global CH_4_ cycle, and that carbon storage generally “wins out” due to the short atmospheric lifetime of CH_4_ (Evans & Gauci, 2023). Nevertheless, any CH_4_ emissions need to be quantified so they can be included in global budgets and models. Mean annual flux was 1.14 g CH_4_ m^−2^ year^−1^ for all vegetated mesocosms, and for the field modelling, which is considerably lower than the IPCC (2014) emission factor of 23.5 g CH_4_ m^−2^ year^−1^ for temperate inland wetlands on mineral soils. As previously mentioned, floodplain meadows will transition between dry grassland and flooded wetland states, and this dynamism was not captured by our study which used static water tables. It is likely that hot moments of CH_4_ emission occur during state changes; flooding in particular is often observed to rapidly stimulate CH_4_ emissions in wetlands and grasslands (Chamberlain et al., 2016; Sánchez‐Rodríguez et al., 2019) but water table drawdown can also enhance CH_4_ emissions under some circumstances due to pressure‐induced degassing and reduced CH_4_ oxidation (Hatala et al., 2012). As such, CH_4_ fluxes measured under floodplain meadow field conditions will likely be more dynamic than fluxes in our mesocosms due to complex nonlinear and asynchronous responses to seasonally fluctuating water tables (Sturtevant et al., 2016). Climate change is predicted to increase the frequency of heat waves and heavy precipitation in north and central Europe (Beniston et al., 2007) which will cause more extreme fluctuations in water tables. Furthermore, these climatic changes will likely drive shifts in plant community composition (Mosner et al., 2015). Together, these changes will have knock‐on effects on CH_4_ emissions, creating feedbacks (Zhang et al., 2023), and may even change some ecosystems from CH_4_ sinks to sources, and vice versa. Currently, there is a lack of baseline data from which to gauge such knock‐on effects. Although some limited data exist for continental European riparian wetlands and grasslands (e.g. Ambus & Christensen, 1995; Audet, Elsgaard, et al., 2013; Audet, Johansen, et al., 2013; Kandel et al., 2019) we are not aware of any data from UK floodplain meadows. In light of the growing number of UK floodplain meadow restoration schemes (Rothero et al., 2020), measurements of CH_4_ are clearly needed in order provided a complete picture of the range of ecosystem services and disservices these ecosystems deliver (Lawson et al., 2018).

## AUTHOR CONTRIBUTIONS

**Mike Peacock:** Formal analysis (lead); investigation (equal); methodology (equal); writing – original draft (lead). **Clare Lawson:** Conceptualization (equal); investigation (equal); methodology (equal); writing – review and editing (equal). **David Gowing:** Conceptualization (equal); supervision (equal); writing – review and editing (equal). **Vincent Gauci:** Conceptualization (equal); supervision (equal); writing – review and editing (equal).

## CONFLICT OF INTEREST STATEMENT

The authors declare no conflicts of interest.

## Supporting information


Data S1.

## Data Availability

Data are available as: Peacock, Mike (2023). Water table depth and plant species determine the direction and magnitude of methane fluxes in floodplain meadow soils. figshare. Dataset. https://doi.org/10.6084/m9.figshare.24105387.v1.
