# Supplementary material for: Water table depth and plant species determine the direction and magnitude of methane fluxes in floodplain meadow soils
Source: Ecol Evol. 2024 Mar 10;14(3):e11147. doi: 10.1002/ece3.11147 (PMC10925825; doi:10.1002/ece3.11147)
Supplement: Supplementary file 1 — Data S1. [file ECE3-14-e11147-s001.docx]

Supplementary Information for the paper:

**Water table depth and plant species determine the direction and magnitude of methane fluxes in floodplain meadow soils**

Mike Peacock^a,b^, Clare Lawson^c^, David Gowing^c^, Vincent Gauci^d,e^.

^a^Department of Geography and Planning, School of Environmental Sciences, University of Liverpool, UK.

^b^Department of Aquatic Sciences and Assessment, Swedish University of Agricultural Sciences, Uppsala, Sweden.

^c^School of Environment, Earth and Ecosystem Sciences, Open University, Milton Keynes, United Kingdom.

^d^Birmingham Institute of Forest Research, University of Birmingham, UK.

^e^School of Geography Earth and Environmental Science, University of Birmingham, UK.

[m.peacock@liverpool.ac.uk](mailto:m.peacock@liverpool.ac.uk)

[michael.peacock@slu.se](mailto:michael.peacock@slu.se)

Supplementary Table 1. Synoptic table showing species composition of the quadrats sampled at North Meadow Cricklade in June 2016. Values represent the number of quadrats (out of five quadrats per treatment) where a species was present, e.g. 5 for *Festuca rubra* in the “Dry” MG4a treatment indicates this species was present in all five of the sampled quadrats. The eleven species in bold were used in the mesocosm experiment (note that *Festuca pratensis* is the only mesocosm species not found in the field plots but is present elsewhere throughout the meadow).

|  |  |  |  |  |  | |  |  |  |  |  |  |  |  |  |  |
| --- | --- | --- | --- | --- | --- | --- | --- | --- | --- | --- | --- | --- | --- | --- | --- | --- |
| **Treatment** | **‘Dry’** | **‘Intermediate’** | **‘Wet’** |  |  |  |  |  |  |  |  |  |  |  |  |  |
| **NVC Community** | **MG4a** | **MG4b** | **MG4d** |  |  |  |  |  |  |  |  |  |  |  |  |  |
| *Festuca rubra* | 5 | 5 |  |  |  |  |  |  |  |  |  |  |  |  |  |  |
| *Ranunculus acris* | 5 | 5 | 2 |  |  |  |  |  |  |  |  |  |  |  |  |  |
| *Holcus lanatus* | 2 | 5 |  |  |  |  |  |  |  |  |  |  |  |  |  |  |
| ***Anthoxanthum odoratum*** | 5 | 5 |  |  |  |  |  |  |  |  |  |  |  |  |  |  |
| ***Sanguisorba officinalis*** | 2 | 5 | 1 |  |  |  |  |  |  |  |  |  |  |  |  |  |
| *Rumex acetosa* | 5 | 5 | 3 |  |  |  |  |  |  |  |  |  |  |  |  |  |
| ***Lathyrus pratensis*** | 4 | 4 |  |  |  |  |  |  |  |  |  |  |  |  |  |  |
|  |  |  |  |  |  |  |  |  |  |  |  |  |  |  |  |  |
| ***Trifolium pratense*** | 5 | 3 |  |  |  |  |  |  |  |  |  |  |  |  |  |  |
| *Lolium perenne* | 5 | 4 |  |  |  |  |  |  |  |  |  |  |  |  |  |  |
| *Cynosurus cristatus* | 4 | 1 |  |  |  |  |  |  |  |  |  |  |  |  |  |  |
| *Taraxacum officinale agg.* | 5 | 3 | 4 |  |  |  |  |  |  |  |  |  |  |  |  |  |
| ***Centaurea nigra*** | 5 | 5 | 1 |  |  |  |  |  |  |  |  |  |  |  |  |  |
| ***Plantago lanceolata*** | 5 | 5 | 3 |  |  |  |  |  |  |  |  |  |  |  |  |  |
| *Trifolium repens* | 2 | 1 | 2 |  |  |  |  |  |  |  |  |  |  |  |  |  |
| ***Prunella vulgaris*** | 5 | 3 |  |  |  |  |  |  |  |  |  |  |  |  |  |  |
| *Bromus racemosus* | 3 | 2 |  |  |  |  |  |  |  |  |  |  |  |  |  |  |
| *Fritillaria meleagris* |  | 1 |  |  |  |  |  |  |  |  |  |  |  |  |  |  |
|  |  |  |  |  |  |  |  |  |  |  |  |  |  |  |  |  |
| *Dactylis glomerata* | 5 | 4 |  |  |  |  |  |  |  |  |  |  |  |  |  |  |
| *Trisetum flavescens* | 1 |  |  |  |  |  |  |  |  |  |  |  |  |  |  |  |
| *Rhinanthus minor* |  | 1 |  |  |  |  |  |  |  |  |  |  |  |  |  |  |
| *Leucanthemum vulgare* | 5 | 4 |  |  |  |  |  |  |  |  |  |  |  |  |  |  |
| *Arrhenatherum elatius* | 4 | 2 |  |  |  |  |  |  |  |  |  |  |  |  |  |  |
| *Heracleum sphondylium* | 3 |  |  |  |  |  |  |  |  |  |  |  |  |  |  |  |
| *Briza media* | 5 |  |  |  |  |  |  |  |  |  |  |  |  |  |  |  |
| *Tragopogon pratensis* | 1 |  |  |  |  |  |  |  |  |  |  |  |  |  |  |  |
| *Linum catharticum* | 3 | 4 |  |  |  |  |  |  |  |  |  |  |  |  |  |  |
| *Ranunculus bulbosus* | 4 |  |  |  |  |  |  |  |  |  |  |  |  |  |  |  |
| *Avenula pubescens* | 3 |  |  |  |  |  |  |  |  |  |  |  |  |  |  |  |
| *Primula veris* | 1 |  |  |  |  |  |  |  |  |  |  |  |  |  |  |  |
|  |  |  |  |  |  |  |  |  |  |  |  |  |  |  |  |  |
| ***Alopecurus pratensis*** | 1 | 5 | 3 |  |  |  |  |  |  |  |  |  |  |  |  |  |
| *Poa trivialis* | 4 | 5 | 3 |  |  |  |  |  |  |  |  |  |  |  |  |  |
| *Deschampsia cespitosa* | 1 | 2 |  |  |  |  |  |  |  |  |  |  |  |  |  |  |
| *Rumex crispus* |  |  | 1 |  |  |  |  |  |  |  |  |  |  |  |  |  |
| *Agrostis stolonifera* | 5 | 5 | 5 |  |  |  |  |  |  |  |  |  |  |  |  |  |
| ***Filipendula ulmaria*** | 4 | 2 | 5 |  |  |  |  |  |  |  |  |  |  |  |  |  |
| *Cardamine pratensis* |  | 1 | 4 |  |  |  |  |  |  |  |  |  |  |  |  |  |
| *Carex acuta* |  |  | 2 |  |  |  |  |  |  |  |  |  |  |  |  |  |
| *Polygonum amphibium* |  |  | 1 |  |  |  |  |  |  |  |  |  |  |  |  |  |
| *Lysimachia nummularia* |  |  | 3 |  |  |  |  |  |  |  |  |  |  |  |  |  |
|  |  |  |  |  |  |  |  |  |  |  |  |  |  |  |  |  |
| *Calliergon cuspidatum* | 1 |  | 2 |  |  |  |  |  |  |  |  |  |  |  |  |  |
| *Trifolium dubium* | 5 |  |  |  |  |  |  |  |  |  |  |  |  |  |  |  |
| *Ophioglossum vulgatum* | 3 |  |  |  |  |  |  |  |  |  |  |  |  |  |  |  |
|  |  |  |  |  |  |  |  |  |  |  |  |  |  |  |  |  |
| *Caltha palustris* |  |  | 3 |  |  |  |  |  |  |  |  |  |  |  |  |  |
| *Carex disticha* |  |  | 4 |  |  |  |  |  |  |  |  |  |  |  |  |  |
| *Carex nigra* |  |  | 1 |  |  |  |  |  |  |  |  |  |  |  |  |  |
| *Equisetum palustre* | 2 |  |  |  |  |  |  |  |  |  |  |  |  |  |  |  |
| *Oenanthe fistulosa* |  |  | 2 |  |  |  |  |  |  |  |  |  |  |  |  |  |
| *Galium palustre* |  |  | 1 |  |  |  |  |  |  |  |  |  |  |  |  |  |
| *Myosotis scorpiodes* |  |  | 2 |  |  |  |  |  |  |  |  |  |  |  |  |  |
|  |  |  |  |  |  |  |  |  |  |  |  |  |  |  |  |  |
| ***Leontodon autumnalis*** | 1 | 1 | 3 |  |  |  |  |  |  |  |  |  |  |  |  |  |
| ***Lotus corniculatus*** | 4 |  | 1 |  |  |  |  |  |  |  |  |  |  |  |  |  |
| *Silaum silaus* | 2 | 3 | 5 |  |  |  |  |  |  |  |  |  |  |  |  |  |
| *Vicia cracca* | 1 | 1 | 3 |  |  |  |  |  |  |  |  |  |  |  |  |  |
| *Phleum pratense* |  | 2 | 5 |  |  |  |  |  |  |  |  |  |  |  |  |  |
| *Ranunculus repens* | 3 | 2 | 4 |  |  |  |  |  |  |  |  |  |  |  |  |  |
| *Hordeum secalinum* | 5 | 1 |  |  |  |  |  |  |  |  |  |  |  |  |  |  |
| *Potentilla reptans* | 3 |  |  |  |  |  |  |  |  |  |  |  |  |  |  |  |
| *Carex hirta* | 1 | 1 |  |  |  |  |  |  |  |  |  |  |  |  |  |  |
|  |  |  |  |  |  |  |  |  |  |  |  |  |  |  |  |  |
| Mean species m^-2^ | 29.8 | 22.8 | 16.8 |  |  |  |  |  |  |  |  |  |  |  |  |  |
|  |  |  |  |  | |  |  |  |  |  |  |  |  |  |  |  |


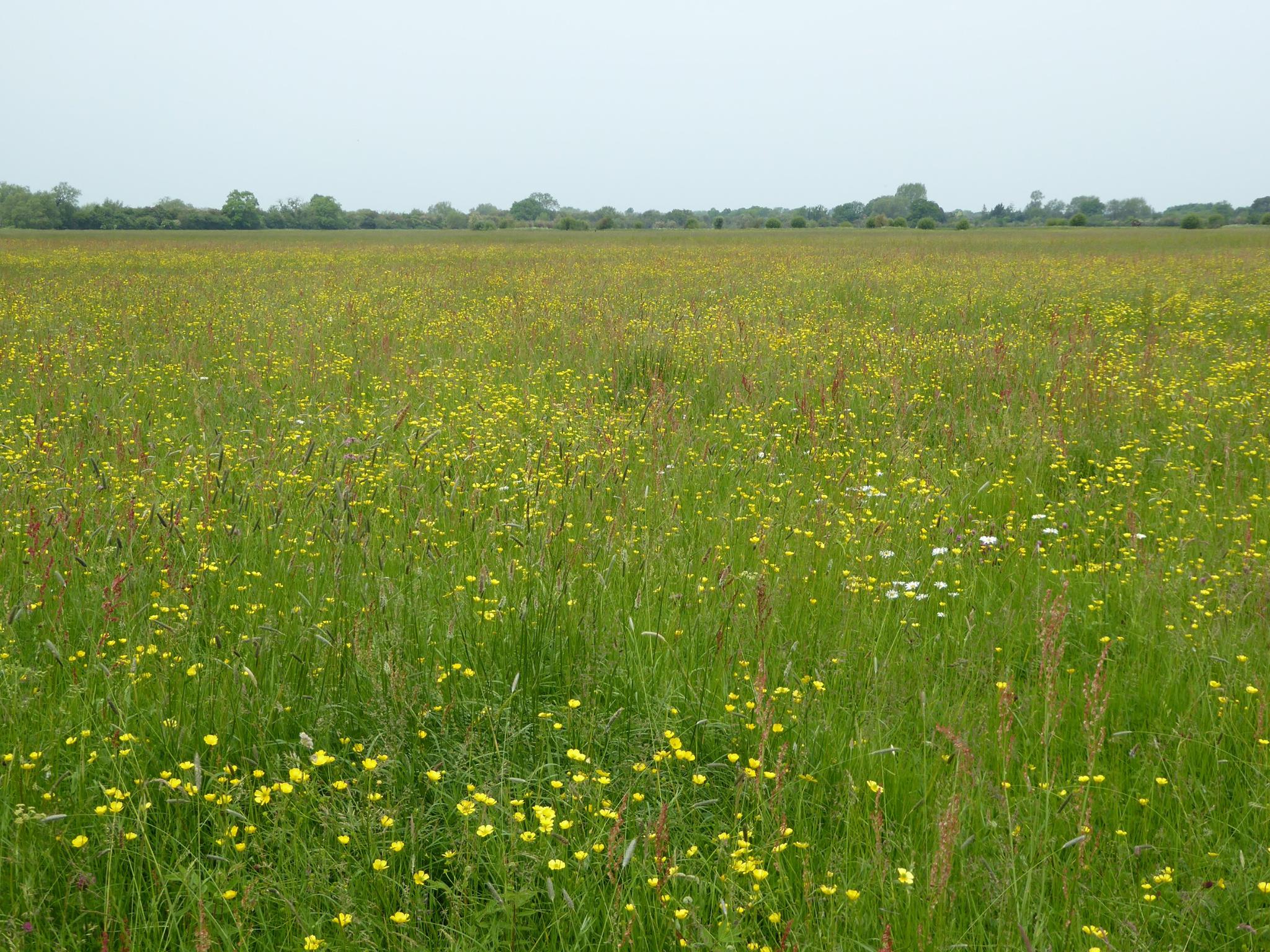


Supplementary Figure 1. A view across Cricklade North Meadow during the June 2016 sampling.


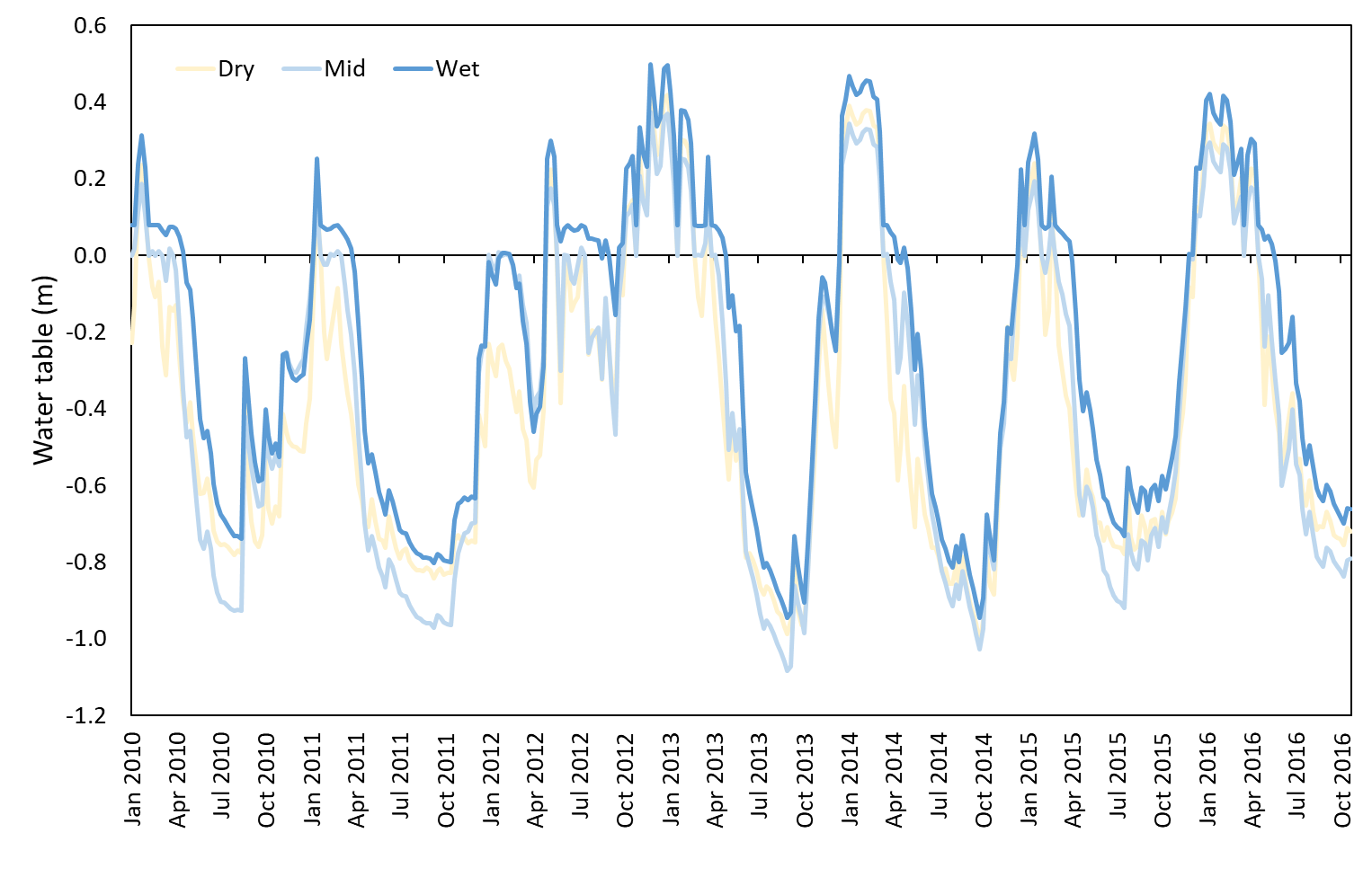


Supplementary Figure 2. Modelled mean water tables for the three locations where CH_4_ fluxes were measured at Cricklade North Meadow. Water table was modelled individually for each of the vegetation quadrats (see Supplementary Table 1) and therefore each water table (dry, mid, wet) is the mean of five time series.


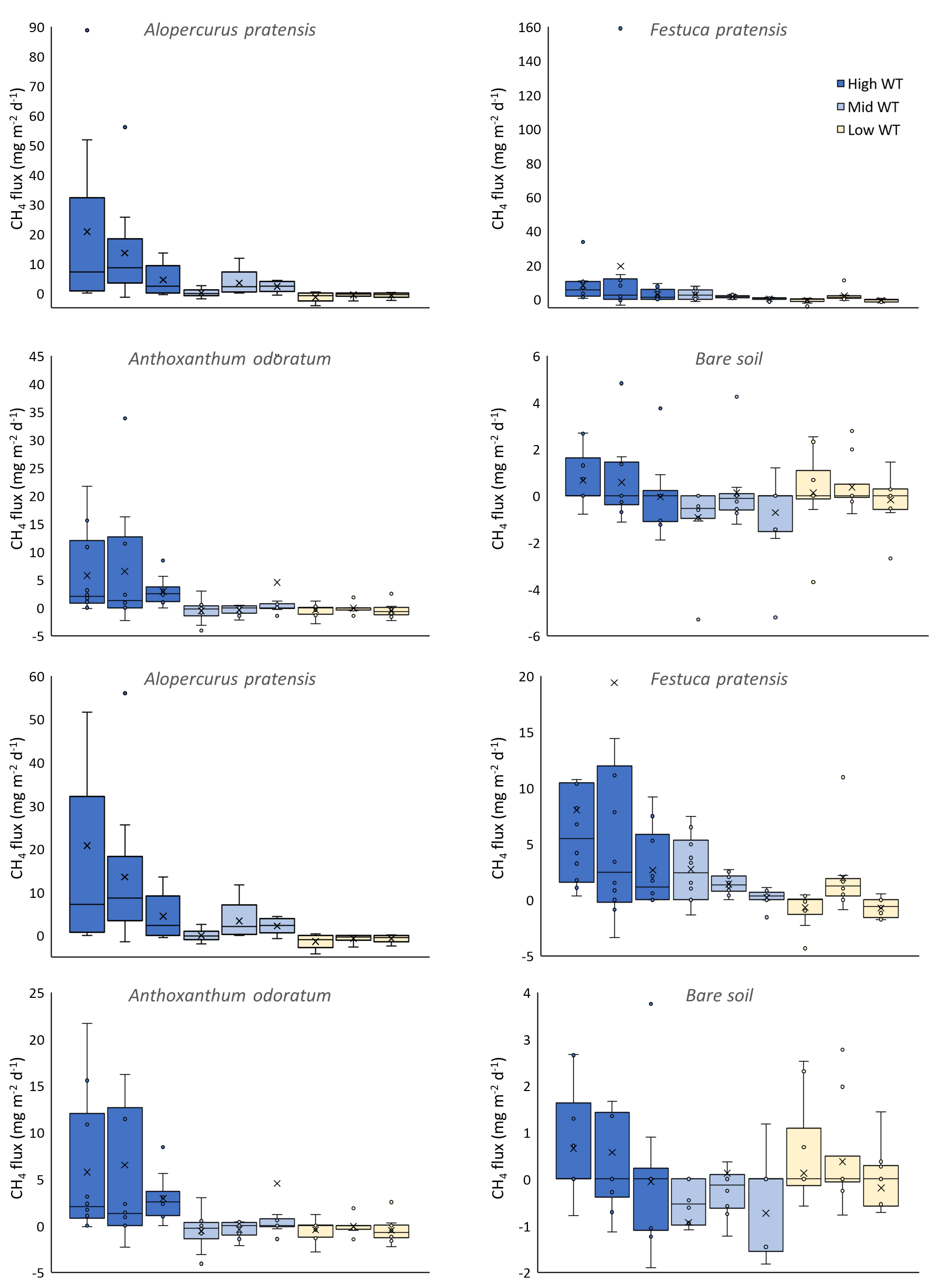


Supplementary Figure 3. Box plot of mesocosm CH_4_ fluxes for all individual mesocosms (n = 36). Each panel shows the mesocosms for each vegetation community (n = 9), and mesocosms are grouped by water table: 15 (high), 30 (mid) and 45 cm (low) below the soil surface. The top four panels show all data, including outliers. The bottom four panels show the same data but with many outliers removed for clarity. Note that y axis scales differ between panels.

Supplementary Figure 4. Scatter plot showing the non-significant correlation between mean air temperature and mean CH_4_ fluxes from vegetated mesocosms in the low water table treatment only (n = 9) for all ten sampling dates.
